# Yeast as a Model to Unravel Mechanisms Behind FUS Toxicity in Amyotrophic Lateral Sclerosis

**DOI:** 10.3389/fnmol.2018.00218

**Published:** 2018-06-28

**Authors:** Michelle Lindström, Beidong Liu

**Affiliations:** ^1^Department of Chemistry and Molecular Biology, University of Gothenburg, Gothenburg, Sweden; ^2^State Key Laboratory of Subtropical Silviculture, School of Forestry and Biotechnology, Zhejiang A&F University, Hangzhou, China; ^3^Center for Large-scale cell-based screening, Faculty of Science, University of Gothenburg, Gothenburg, Sweden

**Keywords:** yeast, ALS, FUS, aggregates, protein toxicity

## Abstract

Fused in sarcoma (FUS) is a multifunctional DNA/RNA-binding protein predominantly localized in the cell nucleus. However, FUS has been shown to accumulate and form aggregates in the cytoplasm when mislocalized there due to mutations. These FUS protein aggregates are known as pathological hallmarks in a subset of amyotrophic lateral sclerosis (ALS) and frontotemporal lobar degeneration (FTLD) cases. In this review, we discussed recent research developments on elucidating the molecular mechanisms behind FUS protein aggregation and toxicity. We mainly focus on studies using the budding yeast (*Saccharomyces cerevisiae*) as a model system, especially on results acquired from yeast genome-wide screens addressing FUS aggregation and toxicity. Human homologs of the FUS toxicity suppressors, identified from these studies, indicate a strong relevance and correlation to a human disease model. By using yeast as a FUS cytotoxicity model these studies provided valuable clues on potential novel targets for therapeutic intervention in ALS.

## Introduction

Amyotrophic lateral sclerosis (ALS), also known as Lou Gehrig’s disease, is a progressive and eventually fatal neurodegenerative disorder with characteristics including loss of upper and lower motor neuron functions, leading to muscular weakness. The cell death seen in the motor neurons of ALS patients occurs with the accumulation of misfolded protein depositions in motor neurons and oligodendrocytes, as well as neuroinflammation (Sreedharan and Brown, 2013). Approximately 10% of all ALS cases are inherited as an autosomal trait (familial ALS, fALS), whereas most cases of ALS are sporadic (sALS). In ALS pathology, certain proteins have been more implicated than others, e.g., SOD1 (Cu-Zn superoxide dismutase 1), TAR-DNA-binding protein-43 kDa (TDP-43) and fused in sarcoma/translocated in sarcoma (FUS/TLS) (Shaw et al., 2001; Guerrero et al., 2016).

The *FUS* gene is located on chromosome 16 and encodes for a 526 amino acid protein which belongs to the family of FET (FUS/EWS/TAF15)/TET (TLS/EWS/TAF15) proteins (Rabbitts et al., 1993; Tan and Manley, 2009). FUS is a DNA/RNA-binding protein with gene regulatory functions including DNA repair, transcriptional control, RNA splicing and mRNA transport to the cytoplasm. FUS shares many similar structures and functions with TDP-43, another DNA/RNA-binding protein, including similar roles in disease induction (Law et al., 2006; Nolan et al., 2016; Ederle and Dormann, 2017).

In many cell types, FUS and TDP-43 are ubiquitously expressed throughout both the cytoplasm and the nucleus and shown to shuttle between these locations. The mechanism behind this shuttling is still not fully explained (Zinszner et al., 1997; Andersson et al., 2008). However, the wild-type FUS protein is predominantly located in the nucleus of neurons and glial cells but when mutated it has been found to accumulate into cytoplasmic aggregates, in synergy with ALS pathology. FUS is thereby lost from the nucleus, most likely resulting in the loss of the normal nuclear protein functions and/or gain of new toxic functions in the cytoplasm (Ling et al., 2013; Ederle and Dormann, 2017). FUS aggregates have been implicated in 7.5% of fALS and <1% of sALS cases, as well as in rare forms of frontotemporal lobar degeneration (FTLD; Kwiatkowski et al., 2009; Vance et al., 2009; Tarlarini et al., 2015). Not only mutated FUS has been implicated in the progression of neurodegenerative diseases, wild-type FUS has also been observed to abnormally aggregate and contribute to a disease phenotype. This has been seen in e.g., most TDP-43- and tau-negative FTLD cases, as well as in some cases of juvenile ALS with basophilic inclusions and a subtype of FTLD-FUS, also with basophilic inclusion bodies (Huang et al., 2010; Urwin et al., 2010; Matsumoto et al., 2015). Furthermore, studies show that FUS strongly interacts with pathological neuronal intranuclear inclusions found in the brains of patients with Huntington disease (HD), spinocerebellar ataxia and dentatorubral-pallidoluysian atrophy (Doi et al., 2010; Woulfe et al., 2010).

### Yeast Models of Neurodegeneration

The simple and well-characterized model organism *Saccharomyces cerevisiae* has been extensively used when studying the mechanisms behind, and impact of protein misfolding and aggregation, typical neurodegenerative disease phenotypes (Outeiro and Lindquist, 2003; Sun et al., 2011). There are many advantages to working with yeast, such as a fully sequenced genome, facilitated genetic manipulations and the existence of a high number (approx. 20%) of orthologous gene families linked to human diseases (Tenreiro et al., 2013). Moreover, many of the complex processes and pathways coupled to protein folding diseases are preserved in yeast and can therefore be studied in this more accessible model (Heinicke et al., 2007). In accordance, *Saccharomyces cerevisiae* contains no FUS homolog, but many cellular pathways coupled to FUS are shared between human and budding yeast cells (Ju et al., 2011).

Yeast gene-to-gene and genome-wide high-throughput screening techniques have played a fundamental and pioneering role in describing protein localization, deletion and over-expression phenotypes as well as identifying modifiers of protein-associated toxicity, thereby contributing to our present understanding of pathways involved in the most prominent human neurodegenerative diseases (Krobitsch and Lindquist, 2000; Outeiro and Lindquist, 2003; Ju et al., 2011; Sun et al., 2011). For instance, yeast models of Parkinson’s disease (PD) and Huntington disease (HD) have uncovered many of the mechanisms behind the toxic aggregation of mutated huntingtin (htt), implicated in HD, and α-synuclein, one of the proteins behind the cytoplasmic inclusions found in PD. It has been shown that α-synuclein induces dose-dependent toxicity, whereas in the case of huntingtin, yeast studies have further validated discoveries indicating that longer polyglutamine stretches in htt increase the tendency of the protein to form insoluble inclusions (Krobitsch and Lindquist, 2000; Outeiro and Lindquist, 2003; Tenreiro et al., 2013). Similarly, yeast genome-wide expression studies focusing on Sen1, the yeast homolog to human Senataxin, implicated in amyotrophic lateral sclerosis 4, have uncovered that mutated SEN1 results in growth defects and increased cellular reactive oxygen species levels (Sariki et al., 2016). Large-scale yeast screenings utilizing gene deletion, and gene over-expression libraries have been frequently employed in neurodegenerative research. For instance, when uncovering that pathways involved in lipid metabolism, vesicular transport and vacuolar degradation harbor proteins that work as potential enhancers and suppressors of α-synuclein toxicity, whereas the kynurenine pathway works as a modifier of htt toxicity (Willingham et al., 2003; Giorgini et al., 2005; Zabrocki et al., 2008). Notably, yeast high-throughput screens are a convenient first approach to the discovery of new mechanisms behind disease progression, followed by validation in more complex animal models and finally a potential discovery of new candidate therapeutic targets (Tenreiro et al., 2013).

**Figure 1:**
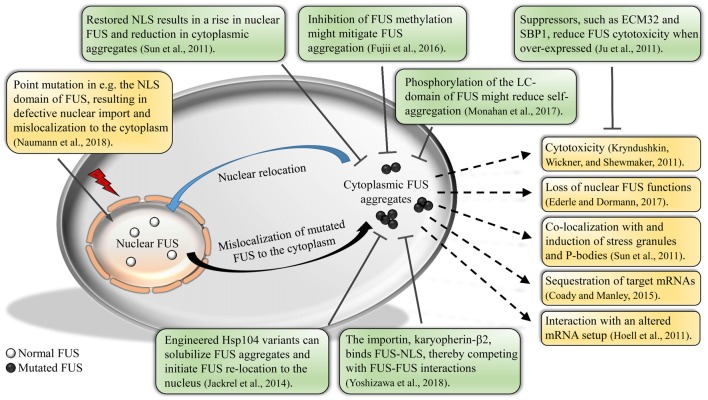
Impact of fused in sarcoma (FUS) mislocalization and aggregation. Normal FUS is predominantly localized to the nucleus. Due to defective methylation of the protein or mutations in the nuclear localization signal (NLS), FUS will mislocalize to the cytoplasm, forming aggregates. Cytoplasmic FUS aggregation could result in various unfavorable outcomes, e.g., cytotoxicity and altered mRNA interactions of FUS. Studies show that a relocation of FUS back into the nucleus is possible by e.g., restoring the NLS or various post-translational modifications, thereby reducing the amount of aggregated FUS and cytotoxicity. Further, studies have uncovered new potential suppressors of FUS cytotoxicity, as well as engineered disaggregases that solubilize aggregated FUS.

In the following, we review recent research developments concerning the DNA/RNA-binding protein FUS and its role in ALS pathogenesis and progression. We focus on research using the yeast system; however, studies in other model systems are also discussed (see outline in Figure 1).

## Domains Required for FUS Aggregation

It has been shown that most FUS mutations coupled to ALS reside in certain distinct domains of *FUS*, such as the N-terminal prion-like domain, including a portion of the glycine-rich region and the nonclassical C-terminal nuclear localization signal (NLS) (Lagier-Tourenne et al., 2010). Other domains present in FUS are the conserved RNA-recognition motif (RRM), the C2/C2 zinc finger motif as well as several arginine-glycine-glycine (RGG)-repeat regions (Burd and Dreyfuss, 1994; Morohoshi et al., 1998; Yoshizawa et al., 2018). The DNA/RNA-binding ability of FUS is enabled through the zinc finger motif, the RRM region, and the RGG regions (Burd and Dreyfuss, 1994; Iko et al., 2004; Ederle and Dormann, 2017). As previously mentioned, a prion-like domain, i.e., a region enriched in polar amino acids (glutamine, glycine, serine and tyrosine, QGSY) has been identified in FUS. These regions are common in RNA-binding proteins and are hypothesized to drive protein aggregation in neurons (Cushman et al., 2010; Gitler and Shorter, 2011; King et al., 2012).

Yeast studies have shown that full-length FUS assemble into multiple cytoplasmic inclusions in yeast (Ju et al., 2011; Kryndushkin et al., 2011; Sun et al., 2011). However, in order to uncover the exact sequence regions and domains required for FUS aggregation and cytotoxicity in *S. cerevisiae*, the aggregation and intracellular location of constructed FUS truncations coupled to a fluorescent tag, have been studied. It has been shown that FUS constructs missing the C-terminal part, including the NLS and the RGG-regions, localize to the nucleus. However, already when only adding back one of the RGG-regions (amino acids 371–422), clear cytoplasmic aggregation of FUS could be seen. In other words, the RGG domain is required for cytoplasmic aggregation of FUS (Sun et al., 2011). Furthermore, constructs containing all domains except the prion-like N-terminal QGSY-region induced only diffuse cytoplasmic staining of FUS with a few small cytoplasmic foci. The QGSY-region of *FUS* is therefore needed for full aggregation to occur but not enough to cause cytoplasmic aggregation in itself (Sun et al., 2011). Studies in insect cells further support the conclusion that the N-terminal prion-like domain is essential for the forming of FUS inclusions (Patel et al., 2015).

A similar approach has also been performed with focus on TDP-43 where it was found that the C-terminal prion-like domain, the region containing most of the ALS-linked mutations in *TDP-43*, was necessary for, and even increase, TDP-43 aggregation and toxicity (Johnson et al., 2008; Cushman et al., 2010). In addition to the C-terminal prion-like domain being necessary for aggregation and toxicity of TDP-43, a portion from RRM2 (RNA recognition motif 2) is also required (Johnson et al., 2008). However, when the entire C-terminal part including the RRM region was deleted in *FUS*, it resulted in nuclear localization of the FUS protein, indicating that this domain is not needed for cytoplasmic aggregation of FUS in yeast (Sun et al., 2011).

In order to establish whether the same FUS domains are needed for aggregation in a mammalian cell model, the previously mentioned full-length FUS and some of the FUS constructs were transfected into COS-7 cells (Sun et al., 2011). In accordance with the yeast results, the FUS deletion constructs missing the C-terminal part, including the RGG region (amino acids 371–422), localized to the nucleus, even though they did not contain the NLS, indicating that other sequences also enable nuclear import of FUS. Furthermore, when adding back the RGG region (amino acids 371–422), FUS aggregated in the cytoplasm of COS-7 cells, as observed in yeast. It has therefore been proposed that domains needed for FUS aggregation in yeast are also needed for aggregation in mammalian cells (Sun et al., 2011). However, in contrast to what has been seen in yeast cells, full-length FUS almost always localizes to the nucleus in mammalian cells, rarely forming transient cytoplasmic foci (Kwiatkowski et al., 2009; Vance et al., 2009; Dormann et al., 2010). Nonetheless, studies show that this is due to differences in the nuclear import process of yeast and mammalian cells (Ju et al., 2011).

Upon stress, phase separation of FUS is important in order for the protein to associate with cytoplasmic stress granules, a normal and reversible process in healthy cells. Studies in mammalian cells have shown that assemblies of FUS are dynamic and turn over quickly as well as always relax into a spherical shape upon fusion, indicators of phase separation into a state called liquid droplets (Patel et al., 2015). Moreover, an atomic force microscopy-infrared nanospectroscopy approach has suggested that FUS liquid-liquid phase separation is guided by cation-π interactions between tyrosine residues in the LC-domain and arginine residues in the RGG domains. The strength of these interactions is regulated by arginine methylation, where hypomethylation of selected FUS arginines has been shown to drive FUS condensation into stable intermolecular β-sheet-rich hydrogel structures (Qamar et al., 2018).

Furthermore, studies indicate that over prolonged time, FUS liquid droplets convert to an aggregated fibrous state, a process hastened by ALS-linked mutations in the prion-like domain of FUS. As previously mentioned, studies in insect cells indicate that the formation of liquid droplets is dependent on the N-terminal prion-like LC-domain of FUS (Patel et al., 2015). In addition, data from solid-state nuclear magnetic resonance methods illustrate the mechanism of the FUS LC-domain in the formation of the FUS fibril cores. The study shows that out of the 214 residues found in the LC-domain, only a 57 residue-segment composes the fibril core while the remainder of the segment is dynamically disordered. The LC-domain of FUS does not display any hydrophobic interactions within the core (Murray et al., 2017).

Protein aggregation and toxicity can be enhanced by certain domains in other aggregation prone proteins in the cell. Such as the yeast protein RNQ1, which contains a Q/N-rich region (glutamines and asparagines). Its aggregated prion form has been reported to significantly enhance aggregation of other Q/N-rich proteins, such as huntingtin (Meriin et al., 2002). No increase in FUS aggregation has been found when the aggregated prion form of RNQ1 is present, in accordance with the small Q/N-region of FUS. However, it has been proposed that the prion form of RNQ1 could enhance the toxicity caused by FUS even though not affecting the aggregation (Park et al., 2018). Furthermore, transgenic mouse models have shown that when wild-type human FUS is over-expressed, the mice develop an aggressive phenotype with increased cytoplasmic expression of FUS in the brain and motor neurons, pathological features seen in ALS and FTLD patients (Mitchell et al., 2013). In addition, transgenic rats over-expressing the wild-type human FUS protein do not show any symptoms at a young age but display cognitive deficiencies when older, due to significant neuronal loss, displaying a resemblance to some ALS and FTLD phenotypes (Huang et al., 2011). These discoveries indicate that over-expression of wild-type FUS is sufficient to promote vast neuron death and thereby aid in augmenting a neurodegenerative disease phenotype (Huang et al., 2011; Mitchell et al., 2013).

To summarize, in order for FUS to form distinct cytoplasmic aggregates in yeast, both sequences in the N-terminal and the C-terminal parts are required. Specifically, the N-terminal prion-like domain and at least the 371–422 RGG domain (Sun et al., 2011; Patel et al., 2015). There are indications that TDP-43 and FUS, even though they are both RNA-binding proteins with many similarities in structure and function, aggregate and develop their disease phenotypes via distinct mechanisms (Johnson et al., 2008). The aggregation pattern of full-length FUS in yeast cells can be of convenience in ALS research, since the physical protein aggregation mimics the FUS aggregation seen in ALS pathology (Kwiatkowski et al., 2009; Vance et al., 2009). Even though the same mutations are not present, the cellular impact of FUS aggregation can still be studied, in addition to being coupled to findings regarding pathological wild-type FUS aggregation.

## FUS Aggregation Causes Cytotoxicity

FUS forms cytoplasmic inclusions when present in yeast but are these aggregates also toxic? Yeast screening assays have identified which human RNA-binding proteins, containing RRMs, aggregate and are toxic at high expression levels in yeast. Out of 132 tested proteins, 35 aggregated and were toxic, including TDP-43 and FUS. Furthermore, some of these proteins also shared the feature of having a prion-like domain. In other words, FUS expression, just like TDP-43 expression, is cytotoxic and correlates with the protein aggregation in the cytoplasm (Kryndushkin et al., 2011; Sun et al., 2011; Park et al., 2018). However, a mouse model expressing a human truncation mutation, associated with early onset ALS (20 years of age), at physiological levels, has been shown to induce adult onset motor neuron loss in the absence of FUS protein aggregates (Devoy et al., 2017). Furthermore, in a *Drosophila* FUS transgenic model, neither wild-type FUS nor ALS-linked FUS mutants formed any cytoplasmic inclusions even though high toxicity was observed. It was suggested that nuclear localization of FUS was necessary for FUS toxicity to occur, whereas the formation of cytoplasmic FUS inclusions was not (Xia et al., 2012).

In accordance with previous studies focusing on sequence domains required for FUS aggregation, the same approach has been carried out to pinpoint which FUS domains are needed for toxicity to occur in yeast. Studies have shown that a decrease in toxicity often occurs with a decline in the aggregation capacity of truncated FUS constructs. When removing segments from either terminal domain of *FUS*, the toxicity is dampened in yeast (Kryndushkin et al., 2011; Sun et al., 2011). Complementary to what has been observed regarding domains needed for FUS aggregation, most of the prion-like N-terminal QGSY-region is also required for enabling FUS toxicity in yeast, but not enough to confer toxicity by itself. Similar results have been achieved with TDP-43 (Sun et al., 2011). For toxicity to occur, the 371–422 RGG domain of FUS seems to be required for FUS cytotoxicity. However, the extreme C-terminal domain with the last 25 amino acids, where most ALS-linked FUS mutations reside, is not necessary for the toxicity effect. When comparing toxicity caused by full-length FUS and FUS constructs missing the last 25 amino acids, it has been shown that the construct is slightly more toxic. In other words, when the N-terminal part and at least the RGG domain (amino acids 371–422) were present, a rise in toxicity could be observed with each add-back at the C-terminal part of FUS (Sun et al., 2011). Furthermore, by disrupting the RNA binding capacity of FUS, a mitigation of FUS toxicity has been seen in yeast but no effect on the cytoplasmic aggregation. This indicates that the RRM region contributes to toxicity in yeast, likely through RNA binding. However, in contrast to what has been seen in TDP-43, adding back the RRM region to the prion-like N-terminal of FUS, does not result in toxicity (Sun et al., 2011).

To summarize, for FUS toxicity to occur in yeast, the prion-like N-terminal, the RRM and the RGG domain (amino acids 371–422) are required, and additional C-terminal sequences are further needed to reach full toxicity (Kryndushkin et al., 2011; Sun et al., 2011). It is still not completely clear whether the FUS misbehavior and consequential ALS pathology are due to a toxic gain or loss of FUS function. Furthermore, the exact role and necessity of FUS aggregation, in the induction of toxicity, is also not yet fully explained. However, when comparing with TDP-43, it seems as if FUS aggregation and toxicity in yeast is carried out in a more complex and multi-domain process, since regions in both the N- and C-terminal parts of FUS are needed for full toxicity.

## Impaired Nuclear Import Results in FUS Aggregation

FUS is a predominantly intranuclear protein. However, during stress, such as heat shock and oxidative stress, FUS can exit and assemble in perinuclear stress granules, and in time re-enter the nucleus. In order for FUS to re-enter the nucleus, the conserved extreme C-terminal NLS of FUS needs to be recognized by a nuclear transport receptor called transportin 1/karyopherin-β2 (Bosco et al., 2010; Dormann et al., 2012).

The importin karyopherin-β2 has been shown to inhibit FUS liquid-liquid phase separation by interacting with and tightly binding the NLS of FUS (Guo et al., 2018; Hofweber et al., 2018; Yoshizawa et al., 2018). Karyopherin-β2 has also been shown to dissolve and reverse phase-separated liquids and fibrillary hydrogels of FUS, by engaging the NLS and LC domains (Guo et al., 2018). Biochemical and nuclear magnetic resonance analysis have further uncovered the occurrence of weak interactions of karyopherin-β2 with multiple other FUS regions, which are also known to promote phase separation. In the proposed model, karyopherin-β2 binds the FUS-NLS with high-affinity, thereby bringing the proteins together, enabling the weak interactions to take place and thereby compete with FUS-FUS interactions, resulting in regulation of FUS inclusion formation and dynamics (Yoshizawa et al., 2018). Moreover, it has been shown that karyopherin-β2, in addition to suppressing FUS phase separation, also hampers the association of FUS with stress granules. However, ALS-linked mutations in the FUS-NLS decreases the chaperone ability of karyopherin-β2, an indication of the further importance of the NLS beyond its role as an import signal (Hofweber et al., 2018).

The FUS-NLS has a non-classical proline-tyrosine (PY) rich domain (PY-NLS). It has been shown that the majority of fALS-associated mutations in *FUS* occur within the NLS, thereby affecting the nuclear import, which correlates with the nuclear import being impaired in some ALS cases. Studies show that FUS is redistributed and recruited into cytoplasmic SGs as a result of an impaired nuclear transport pathway and weakened karyopherin-β2 binding, leading to the production of toxic and insoluble aggregates in neuronal cells (Dormann et al., 2010; Ju et al., 2011; Hofweber et al., 2018). Further studies in human induced pluripotent stem cells derived from motor neurons show that mutations in *FUS*-NLS result in a defective DNA damage response signaling, eventually leading to cytoplasmic FUS mislocalization and aggregation (Naumann et al., 2018).

Yeast also has the same PY-type nuclear localization system as FUS but deviations in the recognition signal results in the mentioned cytoplasmic mislocalization of FUS in yeast, i.e., both ALS-linked mutant FUS and full-length FUS mislocalize to the cytoplasm and are equally toxic in yeast due to a non-functional NLS (Ju et al., 2011). Yeast studies have shown that upon replacement of the non-functional NLS of FUS constructs with a, in yeast, functioning recognition sequence, nuclear localization of FUS can be seen, followed by a reduction in cytotoxicity. In other words, a non-functional FUS-NLS will result in increased toxicity (Ju et al., 2011). This further supports the findings showing that a mislocalization of FUS is essential for the increase in cytotoxicity in yeast (Dormann et al., 2010; Sun et al., 2011). When largely restricting FUS to the nucleus, by fusing a strong heterologous NLS to the N-terminal region of the protein, a considerably lower toxicity level was observed in yeast cells. Even though some cytoplasmic localization of FUS was still present, the now predominantly nuclear FUS resulted in the elimination of cytoplasmic aggregation (Sun et al., 2011). Interestingly, it has been shown that two components, NSR1 and NUP84 (human homologs; NCL and NUP107), related to the nuclear import/export machinery suppress FUS toxicity in yeast when deleted (Sun et al., 2011).

In summary, some ALS-linked FUS mutations have been shown to disrupt the nuclear import process, a behavior resembling the impaired FUS nuclear shuttling seen in some ALS cases, thereby resulting in toxic FUS aggregation in the cytoplasm (Sreedharan and Brown, 2013). However, studies show that when restoring the defective recognition sequence and function of the NLS, FUS relocates back to the nucleus, thereby decreasing the cytotoxicity (Ju et al., 2011; Sun et al., 2011).

## Impact of Post-Translational Modifications on FUS Aggregation

FUS normally becomes arginine-methylated by protein arginine methyltransferase 1 (PRMT1). Extensive dimethylation in the RGG domains of FUS has been found to possibly affect the shuttling and cellular localization of FUS, since dimethylation functions as a signal for nuclear/cytoplasmic translocation of RNA binding proteins (Pahlich et al., 2006; Du et al., 2011; Ju et al., 2011; Fujii et al., 2016). Since the arginine methylations are implicated in the shuttling of FUS, arginine methylation may play a role in the toxicity caused by FUS. Moreover, while inclusions found in ALS-FUS patients contain methylated FUS, it is not present in FTLD-FUS patients (Fujii et al., 2016).

Ju and colleagues explored the possible role of yeast arginine methyl transferases on FUS localization and toxicity upon deletion of either of the major yeast arginine methyl transferases. They found that neither deletion nor over-expression of the arginine methyltransferases, nor the introduction of chemicals known to inhibit the activity of arginine methyltransferases resulted in any effects on FUS aggregation and toxicity levels in yeast (Ju et al., 2011). Another study, in a mammalian cell system, showed that by treating mammalian cells with adenosine dialdehyde, a global methyl transferase inhibitor, the mislocalization and aggregation of FUS mutant was mitigated. Even though this study also showed that excessive treatment with a methylation inhibitor could result in intranuclear aggregation of the FUS mutant, they found that at appropriate levels, inhibition of methylation could mitigate the cytoplasmic mislocalization of the FUS mutant (Fujii et al., 2016). However, in accordance with what has been seen in FTLD patients, other studies have shown that loss of FUS arginine methylation induces liquid-liquid phase separation and stress granule association of FUS, possibly contributing to FUS aggregation. Moreover, in FTLD-FUS patients it has been noted that karyopherin-β2 is aggregated and the arginine methylation needed for FUS-karyopherin-β2 interaction is lost (Hofweber et al., 2018).

As mentioned before, FUS consists of an N-terminal QGSY-rich low-complexity (LC) region (prion-like domain), which has been proposed to drive the formation of reversible liquid droplet structures of FUS (Murakami et al., 2015; Patel et al., 2015). ALS-linked mutations in the LC-domain have been shown to further induce phase transition into irreversible fibrillary hydrogels of FUS, which further induce neurotoxicity in a *C. elegans* model (Murakami et al., 2015). The same domain has also been seen to enable FUS self-assembly in the nucleus of mammalian cells, a critical process required for chromatin-binding and gene regulation. These FUS functions are impaired when ALS-linked mutations are present, such mutations can disrupt the FUS aggregation and subsequent chromatin binding (Yang et al., 2014). Furthermore, when FUS becomes phosphorylated in the LC-domain, the protein’s aggregation-prone behavior becomes mitigated and a disruption of the phase separation is observed. Upon phosphorylation, interactions between LC-domains of FUS proteins can be prevented and thereby hinder any self-aggregation of FUS into pathological inclusions. This change in behavior, which is due to alterations in the biophysical properties of the LC, has been seen in both human and yeast cell models where a subsequent reduction of FUS cytotoxicity was found (Monahan et al., 2017; Murray et al., 2017).

## FUS Induces Stress Granule and P-Body Formation

Stress granules and processing bodies (P-bodies) play vital roles in RNA regulation and processing. RNAs and RNA-binding proteins are incorporated into these structures by highly conserved pathways found in both yeast and humans (Buchan et al., 2008). Heat shock, oxidative stress and other stress situations result in TDP-43 and FUS being localized into these temporary structures, and FUS accumulation in stress granules is a reversible process in healthy neurons (Colombrita et al., 2009; Bosco et al., 2010; Freibaum et al., 2010; Dormann and Haass, 2011). Moreover, mammalian cells studies show that depending on the type of stress the cell is being subjected to, FUS will localize to various compartments, i.e., at DNA lesions upon DNA damage and in stress granules upon heat stress (Patel et al., 2015).

Even though ALS-linked FUS mutants associate with stress granules, FUS was not observed to be significantly associated with P-bodies in mammalian cells, similarly to previous TDP-43 results. ALS-linked FUS mutants displayed increased association with stress granules, contrary to what had been noted for wild-type FUS (Colombrita et al., 2009; Bosco et al., 2010). Furthermore, mutated FUS has been found able to bind and sequester wild-type FUS into stress granules, which might indicate a possible link to its effect on ALS pathogenesis (Guerrero et al., 2016).

In order to test whether stress granule and P-body formations in yeast are coupled to, and perhaps induced by, FUS Sun et al. (2011) expressed yellow fluorescent protein tagged FUS (FUS-YFP) in a yeast model with tagged SG and P-body markers. The study showed that the expression of FUS did indeed induce the formation of both SGs and P-bodies. In addition, FUS physically co-localized with these cellular compartments, just as they do in inclusions of fALS-FUS patients (Sun et al., 2011; Deng et al., 2014). In other words, FUS is able to induce, as well as localize to, RNA granules in yeast, just as in human cells.

During impaired nuclear transport, FUS is recruited into SGs, following redistribution to the cytoplasm (Dormann et al., 2010). Poly-A binding protein 1 (PABP1) was identified as an interacting partner of FUS in mammalian cells. The mutant FUS inclusions co-localized with PABP1-foci, while no such co-localization could be observed between wild-type FUS and PABP1-foci (Gal et al., 2011). In addition, ALS-FUS inclusions have been seen to co-localize with the stress granule marker Ataxin-2, a protein involved in mRNA regulation and stress granule formation, in spinal cord tissue of ALS patients (Elden et al., 2010). It has also been proposed that even though FUS accumulation in stress granules is a reversible and normally occurring phenomenon in healthy neurons, it could advance to harmful aggregation of FUS in stress granules during chronic stress (Ling et al., 2013). The above-mentioned studies provide evidence that stress granules and P-bodies are coupled to FUS functions, indicating an interesting correlation between mutant FUS, stress granules and ALS pathology.

## FUS Mutants Display Altered RNA Interactions

Many FUS functions involve the binding of RNA, including thousands of pre-mRNAs, with a preference for long introns (Fujii et al., 2005). It has been shown that FUS is able to interact directly with RNA via hydrogen bonds and ring stacking. This contact is enabled through the most conserved region of FUS, the RNA-binding domain (Burd and Dreyfuss, 1994). Other domains have also been observed to bind RNA or increase the RNA affinity, e.g., the zinc finger domain and the RGG motifs (Iko et al., 2004). ALS mutations in *FUS* have been seen to affect the expression of its target genes, i.e., by sequestering these target mRNAs within the insoluble cytoplasmic FUS aggregates (Coady and Manley, 2015). In HEK293 cells, ALS-linked human FUS mutants uniquely targeted an overrepresented group of transcripts, originating from endoplasmic reticulum and ubiquitin-proteasome-linked gene types. The mutant FUS variants did not display any impaired RNA-binding capabilities but showed a striking change in binding patterns and an increase in unique targets compared to proteins in the FET family (Hoell et al., 2011). The identification of RNA targets and effects of both mutant and wild-type FUS provides insights into the systems and mechanisms underlying the aggregation and toxicity of FUS.

## FUS Toxicity Suppressors

An interesting question regarding FUS toxicity is whether there are any potential suppressors in the cell that could counteract the toxicity caused by mislocalized FUS. In order to answer this question, Ju and colleagues performed a genome-wide screen using a yeast model (over-expression library), where over 5000 genes were each transformed into a yeast strain expressing an integrated and moderately toxic FUS. The outcome of this screen was the identification of five yeast genes (*ECM32*, *NAM8*, *SBP1*, *SKO1* and *VHR1*) that were able to rescue the FUS toxicity when over-expressed. All the suppressors were DNA/RNA binding proteins, like FUS, and had not been implicated as suppressors of toxicity caused by other neurodegenerative diseases proteins, indicating that these proteins are specific to FUS (Ju et al., 2011).

The identified suppressors were only capable of partially suppressing the toxic FUS effects on yeast growth, and they did not alter the expression level, the location of FUS, or the inclusion formation (Ju et al., 2011). Out of the five screen hits, extracellular mutant 32 (ECM32) was found to have a human homolog, human up-frameshift protein 1 (hUPF1), which has been shown to be involved in mRNA quality control and surveillance, and also found to localize to P-bodies and cytoplasmic granules (Ohnishi et al., 2003; Isken and Maquat, 2008; Ju et al., 2011). Further study showed that the over-expression of *hUPF1*, and of *hUPF2* (physical interaction partner of *hUPF1*), also rescued FUS toxicity in yeast (Ju et al., 2011). The potential mechanism underlying the rescue ability of these expressed genes probably involves compensation or restoration of essential cellular functions disturbed by the FUS toxicity. One such possible disturbance could be that the deviance in FUS behavior results in RNA and/or other molecules become sequestered, thereby disrupting normal RNA functions. For instance, hUPF1 plays a vital role in mRNA surveillance and RNA quality control, functions that might enable the rescue seen here (Isken and Maquat, 2008; Ju et al., 2011).

When conducting a similar genome-wide yeast over-expression screen, Sun et al. (2011) found 24 suppressors and 10 enhancers of FUS toxicity, including the same five suppressor hits also identified by Ju et al. (2011). The largest class of genes uncovered in the screen included RNA-binding proteins, in accordance with the results by Ju et al. (2011), and proteins involved in RNA metabolism. Moreover, three stress granule components, translation initiation factors (Tif2 and Tif3) and Pab1, were isolated as FUS toxicity suppressors. Pab1 is involved in stress granule formation in yeast and has a human homolog, called PABP1, mentioned earlier as a FUS-interactor in mammalian cells (Gal et al., 2011). This indicates that stress granule components might play a key role in reducing FUS toxicity.

It has been shown that over-expression of FUS in yeast results in toxicity as well as inhibition of the ubiquitin proteasome system. This phenotype can be relieved by over-expression of the heat shock protein 40 chaperone, Sis1. This was done without altering the FUS levels in the yeast cells (Park et al., 2018). In accordance, Jackrel et al. (2014) conducted a study to test the effect of protein disaggregases when attempting to eliminate misfolded toxic protein conformers. They aimed at engineering heat shock protein 104 (Hsp104) variants that would reverse the protein misfolding seen in neurodegenerative disorders. The modifications to Hsp104, a conserved hexameric AAA+ protein disaggregase from *S. cerevisiae*, would enhance Hsp104 and thereby eliminate substrates implicated in ALS (Jackrel et al., 2014; Torrente et al., 2016). Under normal conditions, Hsp104 solubilizes disordered aggregates and amyloids, restoring the native protein conformation, but displays very limited activity against human neurodegenerative disease proteins. Upon *HSP104* gene deletion, no changes in FUS aggregation nor toxicity has been observed (Shorter, 2008; Vashist et al., 2010; Kryndushkin et al., 2011). Jackrel and colleagues developed yeast platform methods which enabled them to screen large libraries of Hsp104 variants for abilities in suppressing toxicity caused by protein misfolding. The result was a series of reprogrammed and enhanced Hsp104 variants that not only reversed FUS aggregation and toxicity but also restored correct FUS localization, thereby restoring proteostasis. Furthermore, engineered Hsp104 variants have been shown to also mitigate neurodegeneration in a *C. elegans* model (Jackrel et al., 2014).

The suppressors identified in these genome-wide screens illuminate the cellular pathways linked to abnormal FUS aggregation and thereby propose new potential therapeutic targets.

## Concluding Remarks

Although the simple yeast system does not display all of the cellular processes found in human cells, many essential cellular functions are shared. Conveniently, several pathways associated with neurodegeneration are conserved between yeast and humans and have therefore been crucial when establishing yeast as a model for protein mislocalization and aggregation in neurodegenerative diseases. Despite some clear differences between yeast and mammalian cell functions, such as wild-type FUS locating to the cytoplasm or the nucleus, the yeast system has proven to be a highly useful model of FUS cytoplasmic aggregation and toxicity, critical pathological events in ALS. In accordance, the yeast studies discussed in this review have uncovered several conserved molecular mechanisms behind FUS protein misfolding, such as sequence domains required for aggregation and toxicity, as well as subsequent impact on other cellular pathways, such as RNA metabolism and stress granule formation. Studies show that the toxicity caused by FUS could be a result of misfolded proteins surviving the protein control system and consequentially disturbing the normal cellular functions. Such a disturbance might be a sequestration of proteins and/or RNAs by the cytoplasmic aggregation of FUS, thereby displaying a gained toxic ability. But also, the loss of normal FUS functions in the nucleus, such as RNA processing, imply a loss of function affecting the cell. However, there are studies indicating that cytoplasmic aggregation of FUS might not at all be needed for FUS toxicity to occur in mammalian systems.

Moreover, yeast has served as an optimal platform for isolating FUS toxicity suppressors from large libraries, and uncovered possible candidate genes with human homologs, which strongly indicate that yeast can serve as a proper model for studying FUS cytotoxicity. Further developing genome-wide genetic and phenomic approaches can be used to address remaining challenges in understanding the FUS-ALS pathogenesis, such as identification and characterization of the altered protein and RNA interactions affected by FUS mutants, as well as characterizing the genetic susceptibility and environmental triggers of the disease. By identifying the molecular pathways underlying ALS and the role of external environmental factors in disease development, new therapeutic approaches and disease prevention methods could be uncovered.

## Author Contributions

BL conceived and outlined the paper. ML wrote the manuscript with the help from BL.

## Conflict of Interest Statement

The authors declare that the research was conducted in the absence of any commercial or financial relationships that could be construed as a potential conflict of interest.
